# Systematic review and standardised assessment of Chinese cross-cultural adapted hip Patient Reported Outcome Measures (PROMs)

**DOI:** 10.1371/journal.pone.0257081

**Published:** 2021-09-20

**Authors:** James Reeves Mbori Ngwayi, Jie Tan, Ning Liang, Emmanuel Gildas Eric Sita, Kenedy Uzoma Obie, Daniel Edward Porter

**Affiliations:** 1 School of Clinical Medicine, Tsinghua University, Beijing, China; 2 School of Clinical Medicine, Fujian Medical University, Fujian, China; 3 School of Clinical Medicine, Central South University, Changsha, China; 4 Department of Orthopedics, Beijing Huaxin Hospital, Clinical Medicine School, Tsinghua University, Beijing, China; Istanbul University Istanbul Faculty of Medicine: Istanbul Universitesi Istanbul Tip Fakultesi, TURKEY

## Abstract

**Purpose:**

To perform a systemic literature search to identify Chinese cross culturally adapted and new designed Patient Reported Outcome Measures (PROMs) used for hip assessment, then a standardized evaluation of available instruments in order to provide evidence of high-quality PROMs for clinical use and adoption in future hip registries.

**Methods:**

A Systematic Review of the following databases: PUBMED, CINAHL, EMBASE, CNKI was performed to identify relevant PROMs. Instruments underwent standardized assessment and scoring using the EMPRO tool by two independent reviewers. Inter-rater reliability was assessed using intra-class correlation coefficients (ICC).

**Results:**

2188 articles were retrieved, with seven articles fitting the inclusion criteria consisting of six hip PROMs. Five PROMs were cross culturally adapted and one was originally designed in Mandarin Chinese. Total scores (/100) after EMPRO evaluation: Osteoarthritis of Knee and Hip Quality of Life (OAKHQOL): 55; Copenhagen Hip and Groin Outcome Score (HAGOS): 52; International Hip Outcome Tool (SC-iHOT-33): 45; Hip Disability and Osteoarthritis Outcome Score (HOOS): 37; Questionnaire on the Perceptions and Functions of Patients about Total Hip Arthroplasty (QPFPTHA): 36; Oxford Hip Score (OHS): 35. ICC values were 0.73 for the SC-iHOT-33 and ranged between 0.83–0.93 for the other PROMs indicating good to excellent inter-rater agreement.

**Conclusion:**

Among the commonly used hip-specific PROMs found in arthroplasty registries, none of the Chinese adapted versions evaluated by EMPRO is currently rated acceptable for clinical use. Only OAKHQOL and HAGOS reached acceptability threshold. Further research on the attributes of cross-cultural adaptation, interpretability and burden assessment would be helpful.

## Introduction

Hip arthroplasty constitutes one of the most cost-effective interventions in medicine [1]; reported cost-effectiveness ratios based on quality of life years(QALY) for common procedures include: £1372 for hip arthroplasty; £2101/QALY for knee arthroplasty; £3129-6904/QALY for tonsillectomy with adenoidectomy; £4928/QALY for inguinal hernia repair; £13205/QALY for laparoscopic cholecystectomy [2]. Patient Reported Outcome Measures (PROMs) assist in QALY calculation and are typically clinical outcome tools which give a voice to patients, documenting their own perspective without clinician interpretation [3–5]. PROMs increasingly dominate clinical outcome appraisal following advocacy of the central role of patients in their own health care affairs. A variety of hip related PROMs have been developed and are already in worldwide use [6, 7]. Most were originally designed in English. Before their deployment in another linguistic setting they must undergo rigorous transcultural adaptation and translation following guidelines such as International Society for Pharmacoeconomics and Outcomes Research(ISPOR) [8]. As with the English language original versions, implementation of culturally adapted PROMs should follow a quality control process for clinical use. Instruments to assess methodological quality of Health-related Patient Reported Outcomes(HR-PROs) include the Consensus Based Standards for the Selection of Health Measurement Instruments (COSMIN) checklist [9, 10]; and the Evaluating the Measurement of Patient-Reported Outcomes (EMPRO) tool, capable of both qualitative and quantitative assessment of PROs [11].

Arthroplasty registries around the world have expanded their endpoints from failure events such as revision surgery, infection and dislocation to include subjective endpoints such as pain relief, increased functionality and quality of life, involving a shift towards adopting PROMs within the protocol for clinical assessment [12]. Although there are no national or provincial arthroplasty registries in mainland China, it is important to evaluate and document the quality of PROMs presently in use to provide appropriate tools for future registry development. More immediately, surgeons should be aware of the best quality PROMs available for use in their patient population. The aim of the study was to carry out a systematic literature search for Chinese cross-culturally adapted PROMs and new Chinese-designed PROMs for use in the Chinese patients with hip disorders and evaluation using the EMPRO assessment instrument.

## Methods

### Systematic literature review and identification of hip studies

A search was performed of the earliest records up to 22/08/2020 according to guidelines of the Preferred Reporting Items for Systematic Reviews and Meta-Analyses (S1 Checklist) [13]. The following databases were used: PubMed/MEDLINE, EMBASE(OVID), CINAHL(EBSCO) and CNKI (mainland Chinese database). Sensitive filters composed of MeSH terms and keywords based on previously documented PROM search strategies [14–16] (S1 File), were further tailored to fit the target body region and population (Chinese) then applied to the databases. Publication languages for the articles were English and Chinese.

Based on Population, Intervention, Comparison, Outcome (PICO) criteria the following inclusion criteria were adapted: 1. Cross culturally adapted and translated hip PROMS tested in the Mainland Chinese population. 2. Hip specific PROMs evaluating hip procedures 3. PROMs restricted to the Chinese Mainland in Mandarin Chinese.

Exclusion Criteria were: 1. Non-Mainland Chinese PROMs including Mandarin language studies from geographical Taiwan and Hong Kong. 2. Instruments tested on populations outwith Mainland China. 3. Articles not meeting inclusion criteria.

**Fig 1:**
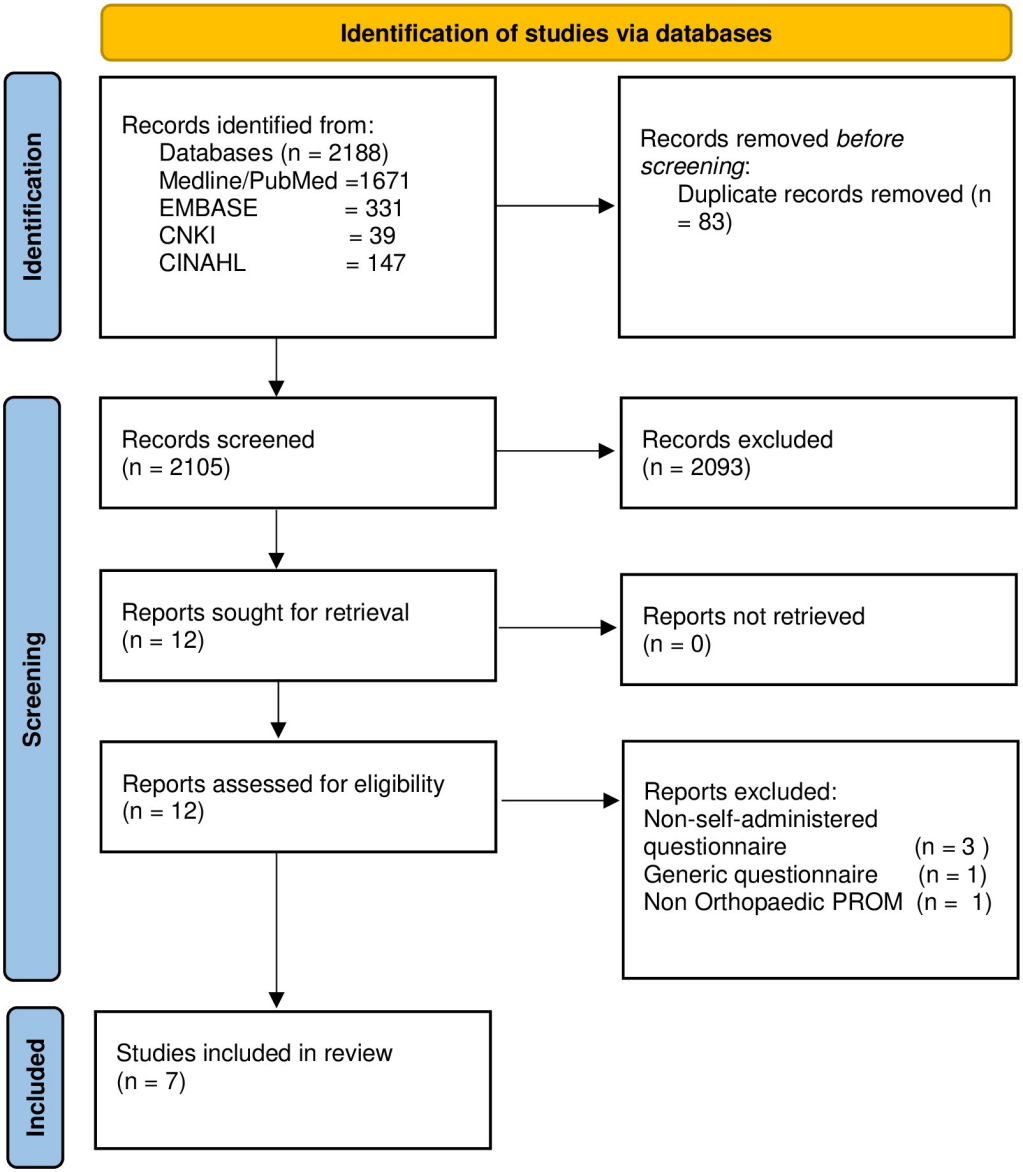
PRISMA flow chart—Systematic literature search.

Screening was carried out as a three-step process (Titles, Abstract and Full texts), performed independently by two reviewers (NJRM, DEP). Outputs were compared and consensus reached. After full text screen and identification of suitable articles we manually reviewed the in-article reference lists for potential relevant articles missed during the electronic search. The PRISMA chart of the review process is illustrated in Fig 1.

### The EMPRO tool

The Evaluation Measurement of Patient Reported Outcomes (EMPRO) tool was designed in 2008 by Valderas JM et al, aimed at qualitative assessment of the methodology and development process of PROs. It comprises 8 attributes, namely: conceptual and measurement model (7items), cross-cultural and linguistic adaptations (3 items), reliability (8 items), validity (6 items), responsiveness to change (3 items), interpretability (3 items), burden (7 items), alternative modes of administration (2 items); in total 39 items [11]. Quantitative assessment is achieved via an inherent scoring system where each item is graded on a 4-point Likert scale, from 4 (strongly agree) to 1 (strongly disagree) with additional option boxes for “No information” and “Not applicable” where necessary.

The questionnaire terminates with a rationale for recommendation by the reviewer according to the following response scale: “Strongly recommended”, “Recommended with provisos or alterations”, “Would not recommend” and “Unsure”.

The EMPRO tool requires a license application via the portal www.bibliopro.org, which is free to-use, and presently available in two languages (English and Spanish). EMPRO has been used before in the systematic assessment of orthopedic-specific shoulder PROMs [17].

### Standardized and systematic evaluation; scoring and analysis

After the systematic review, data pertaining to the measurement properties of the selected PROMs studies were extracted from relevant articles. A standardized assessment of the adequacy of their measurement properties was undertaken using the EMPRO tool. To avoid possible bias due to evaluating cross cultural adaptation across different populations, assessment was restricted to studies on Chinese populations [14]. According to recommendations from the designers, two reviewers (both clinicians with a background in PROMs research) performed the assessment. Both had also completed the online EMPRO training webinar (https://www.isoqol.org/category/webinar/page/3/). The assessment was carried out in two phases. The first phase consisted of each reviewer independently scoring article(s) supporting each cross-culturally adapted hip PROM for methodological attributes, as well as the article describing the original design of the PROM for the conceptual and measurement model assessment. The second phase which followed a consensus method recommended by the EMPRO designers involved discussions between reviewers on discrepancies to obtain a common score for each item. Reviewers were based in two continents and did not converse on scoring until the discussion phase.

Scoring of the methodological attributes was calculated based on developers’ instructions; https://www.isoqol.org/?s=Empro. Specifically, attribute-specific scores are obtained by calculating the response mean of the applicable items when at least 50% of them are rated; and items check marked with the option ‘‘no information” are assigned a score of 1 representing the lowest possible score. The response means for each attribute are then linearly transformed to a range of 0 to 100 (worst to best). Global scores (based on metric properties) are only calculated when at least 3 attributes can be scored and attributes without information are imputed zero. Panoramic assessment (which includes all culture/language versions of the instrument) involves conceptual model, reliability, validity, responsiveness and interpretability while culture/language specific evaluation involves conceptual model, reliability, validity, responsiveness and interpretability and cross-cultural adaptation. Global scores for each domain of at least 50 (/100) are considered acceptable based on previous assessments by Garin O et al. [18].

Data synthesis involves combining results from different studies on measurement properties of the instrument under consideration. Results of measurement properties can differ depending on sample characteristics and setting; thus (dis)similarities have to be appropriately addressed before deciding whether articles contain measurement property data which can be pooled [19]. After the first phase scoring by reviewers, agreement between them was assessed by using two-way, random, single unit, absolute agreement intra-class correlation coefficients ICC [20]. The degree of reviewer agreement was categorized based on Cicchetti (1994) ICC<0.40 poor; 0.40 to 0.59 moderate; 0.60–0.74 good; 0.75–1.00 excellent [21].

Statistical analysis, attribute scores calculation and graphics were performed with Microsoft Excel 2003^®^ (Microsoft, Redmond, WA, USA), Intraclass correlation (ICC) was calculated using SPSS^®^ Version 20.0 software (SPSS Inc, Chicago, IL, USA).

## Results

The literature search procedure resulted in a total of 2188 papers. Articles per database were as follows: PubMed/MEDLINE 1671 (76.4%), EMBASE 331 (15.1%), CINAHL 147 (6.7%) and CNKI 39 (1.8%). A total of seven papers were retained at the end of the process representing six hip-specific instruments. Five of the instruments were cross-cultural adaptations: Copenhagen Hip and Groin Outcome Score (HAGOS); International Hip Outcome Tool (SC-iHOT-33); Osteoarthritis of Knee and Hip Quality of Life (OAKHQOL); Oxford Hip Score (OHS); Hip Disability and Osteoarthritis Outcome Score (HOOS); and one instrument was Chinese-designed: Questionnaire on the Perceptions and Functions of Patients about Total Hip Arthroplasty(QPFPTHA). All papers were published between 2010 and 2019, two questionnaires were unidimensional while four had dimensions ranging from 2–6 dimensions. One PROM was validated for both knee and hip arthroplasty (OAKHQOL). Table 1 describes general characteristics of each Chinese Cross-cultural adaptation and translation of hip PROM.

**Table 1:** General characteristics of Chinese cross-cultural adaptation and translation of hip PROMs.

Questionnaire	Author—Year	Population Characteristic	Dimensions and (items)	Scale design
Copenhagen Hip and Groin Outcome Score (HAGOS) [22]	Shi Qi Cao et al (2018)	Developmental dysplasia of hip joint, osteonecrosis of the femoral head, hip osteoarthritis for THA	6 Domains; symptoms (7 items), pain (10 items), function in ADL (5 items), function in Sport/Rec (8 items), participation in physical activities (2 items), hip- and/or groin-related QoL (5 items).	Standardized from 0 (worst health status) to 100 best health status
		Mean age 64 years		
International Hip Outcome Tool (SC-iHOT-33) [23]	D.H. Li et al (2016)	Avascular necrosis, Hip dysplasia, Femoro-acetabular impingement syndrome for THA	4 Domains; symptoms and functional limitations (16 items, Sport/Rec activities (6 items) job-related concerns (4 items) social, emotional, and lifestyle concerns (7 items).	Standardized from 0 (worst health status) to 100 best health status
		Mean age 43 years		
Osteoarthritis of Knee and Hip Quality of Life (OAKHQOL) [24]	W. Wang et al. (2016)	Osteoarthritis for both hip and knee Arthroplasty	5 Domains; physical activities (16 items), mental health (13 items), pain (4 items), social support (4 items) and social activities (3 items). The rest are three independent items related to relationships, sexual activity and professional life.	Standardized on a scale from 0 (worst quality of life) to 100 (best quality of life)
		Mean age 63 years		
Oxford Hip Score (OHS) - 2 articles [25, 26]	Wei Zheng et al (2013)	Hip OA for THA	12-items	0 (worst) to 48 (best).
		Mean age 66 years		
	Xia Zhen-lan et al (2012)	THA Pre-operation, 1-year post THA and 3-years post THA		
		Mean age 55 years		
Hip disability and osteoarthritis outcome score (HOOS) [27]	X. Wei et al (2012)	Primary hip OA for THA	5 Domains; pain (10 items), other symptoms (10 items), function in (ADL (17 items), function in Sport/Rec (4 items), hip-related QoL (4 items).	Normalized scores, from 0 (indicating extreme symptoms) to 100 (indicating no symptoms),
		Mean age 51 years		
Questionnaire on the perceptions and functions of patients about Total Hip Arthroplasty [28]	Tang Hong Yuan et al (2010)	Femoral head necrosis, emoral neck fracture, hip OA, hip joint dysplasia, AS, Bone tumours, hip joint tuberculosis for THA	10 items	10 (worst) to 50 (best).
		Mean age 57 years		

THA: Total Hip Arthroplasty; OA: Osteoarthritis; AS: Ankylosing spondylitis; QOL: quality of life; ADL: activities of daily living; Sport/Rec: sports and recreation.

Qualities of measurement property domains/attributes for each hip PROM are illustrated in Table 2. Following precedent in other studies [17] the global score for each domain was transformed into a five-point scale (denoted—/ + / ++ / +++ / ++++). +: EMPRO score < 25; ++: EMPRO score 25–49; +++: EMPRO score 50–74; ++++: EMPRO score 75–100; -: EMPRO score not applicable or not calculable according to designer instructions.

**Table 2:** Standardized assessment of measurement attributes of Chinese cross-culturally adapted PROMs using EMPRO.

PROM	Concept and measu-rement model	Cultural and Language Adaptations	Reliab-ility	Vali-dity	Respon-siveness	Interp-retability	Bur-den	Alternative forms of Admin-istration	Total Score /100
OAKHQOL	++++	+++	+++	++++	+++	-	+	-	54.84
HAGOS	+++	+++	+++	++++	+++	-	-	-	52.19
SC-iHOT-33	+++	-	+++	+++	+++	-	++	-	45.05
HOOS	+++	-	+++	+++	+++	-	++	-	36.98
QPFPTHA	+++	-	++	+++	++	-	+	-	36.02
OHS	++	-	+++	+++	+++	-	+	-	35.19

Copenhagen Hip and Groin Outcome Score (HAGOS); International Hip Outcome Tool (SC-iHOT-33); Osteoarthritis of Knee and Hip Quality of Life (OAKHQOL); Oxford Hip Score (OHS); Hip disability and Osteoarthritis Outcome Score (HOOS); Questionnaire on the Perceptions and Functions of Patients about Total Hip Arthroplasty (QPFPTHA);

+: EMPRO score < 25; ++: EMPRO score 25–49; +++: EMPRO score 50–74; ++++: EMPRO score 75–100; -: EMPRO score not applicable or not calculable.

**Fig 2:**
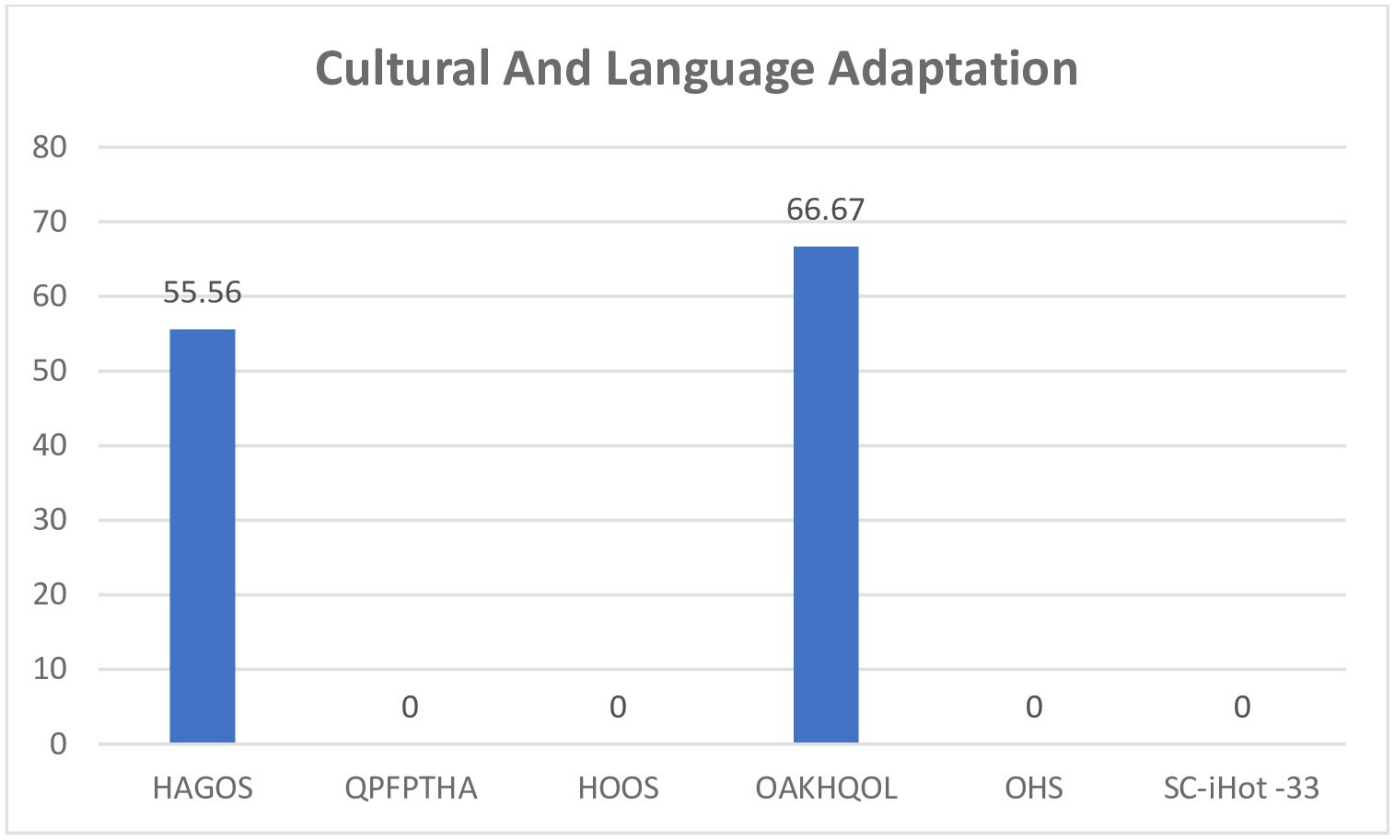
Global attribute score for each PROM as evaluated by EMPRO.

**Fig 3:**
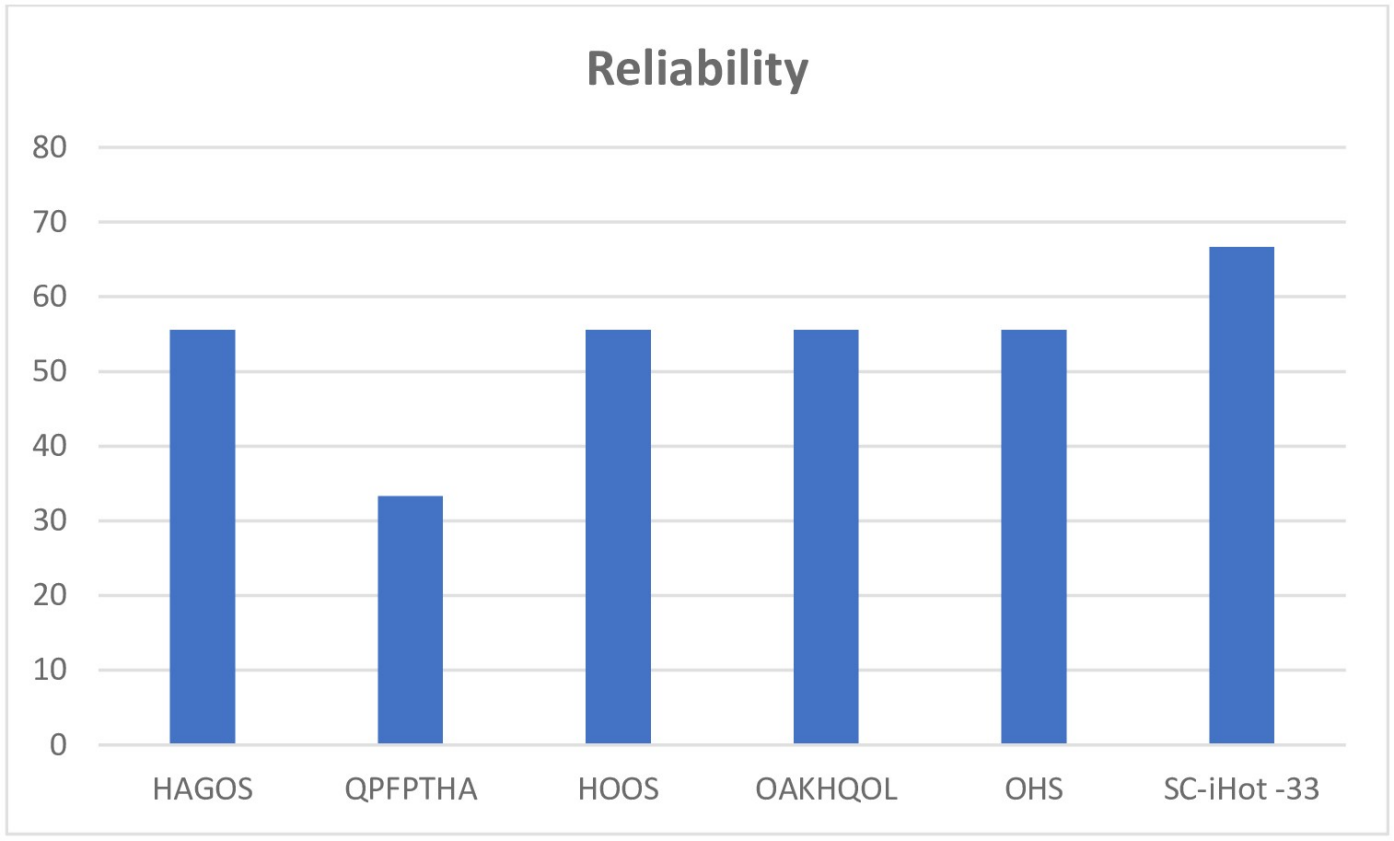
Cultural and language adaptation attribute score for each PROM as evaluated by EMPRO.

**Fig 4:**
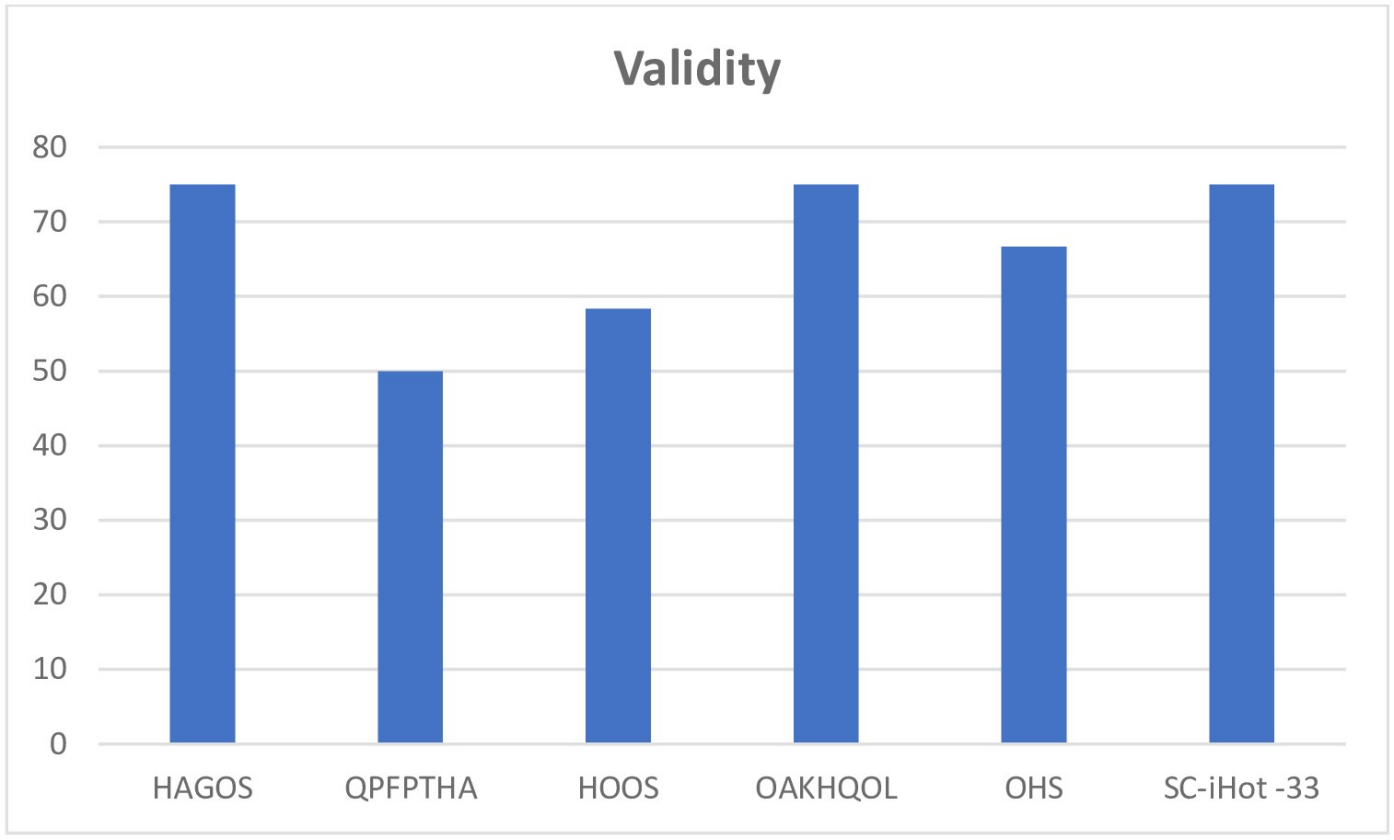
Reliability attribute score for each PROM as evaluated by EMPRO.

**Fig 5:**
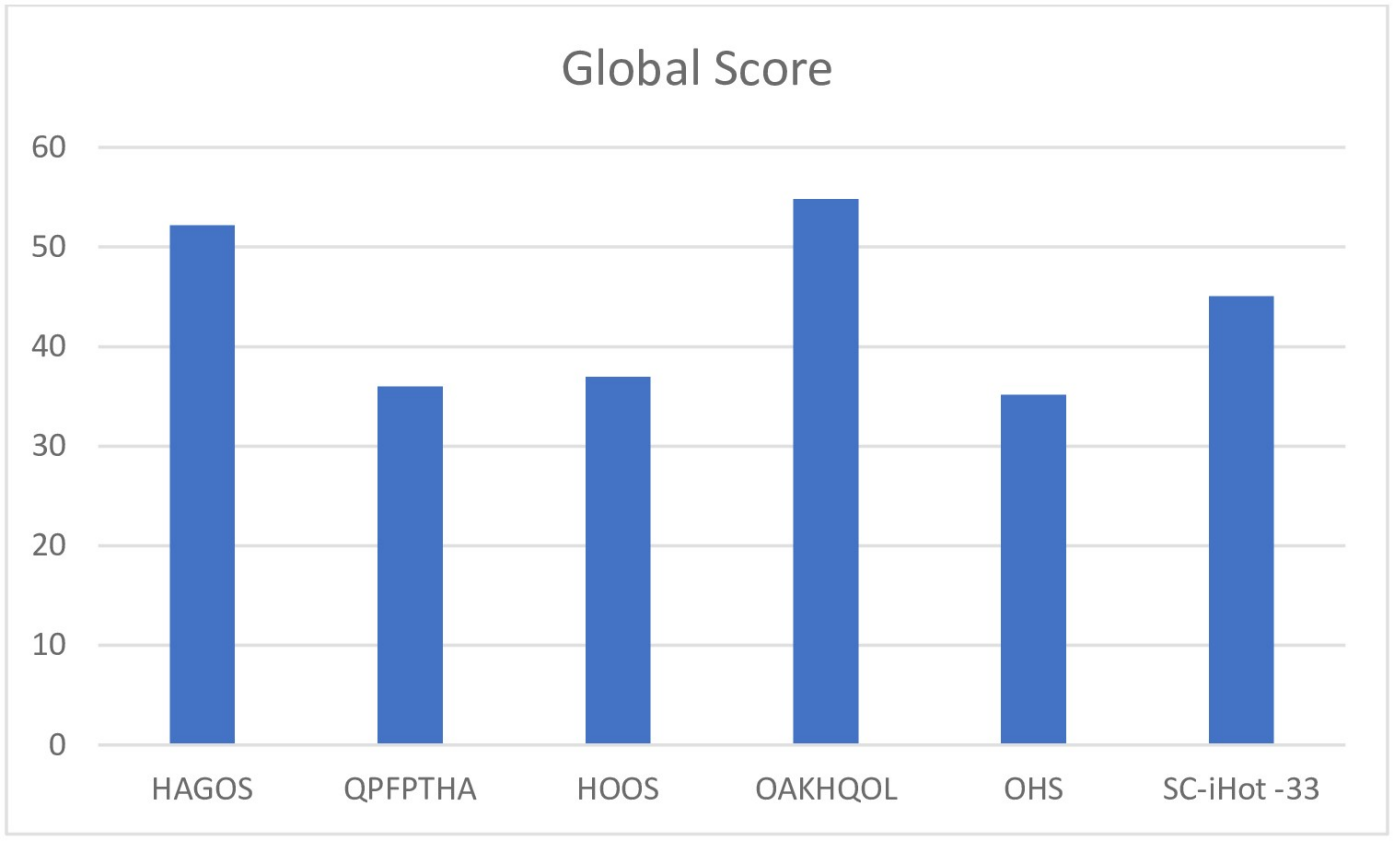
Validity attribute score for each PROM as evaluated by EMPRO.

The properties of each PROM were assessed and scored with the EMPRO tool, for each of the attributes as well as a total score for the PROM. All questionnaires had acceptable scores for the concept and measurement model except OHS which scored ++. Questionnaires were all evaluated for cultural and language adaptations except the QPFPTHA originally designed in simplified Chinese. The HAGOS and the OAKHQOL had acceptable scores while the others could not be calculated (Fig 2). All instruments had acceptable scores on assessment of reliability (Fig 3) and responsiveness except the QPFPTHA which scored ++ in these attributes. On evaluation of validity all scores were above threshold for acceptability (Fig 4); however, data was insufficient for calculating interpretability. Burden scores could not be calculated for HAGOS, and for all remaining PROMs scored below acceptability threshold. Total scores were calculated for each PROM; OAKHQOL and HAGOS had scores above acceptability threshold (54 and 52 respectively) while SC-iHOT-33 scored 45, HOOS scored 36, QPFPTHA scored 36 and OHS scored 35 (Fig 5).

Intra-class correlation coefficient (ICC) values for the assessment of each PROM are depicted in Table 3. The agreement between the two reviewers ranged between ICC = 0.83–0.93 except for the SC-iHOT-33 with ICC of 0.73.

**Table 3:** Inter-rater reliability for 7 articles describing PROMs.

PROM	Intra-class correlation coefficient (ICC)
OHS(zheng et al)	.934
OHS(Xia et al)	.841
OAKHQOL	.856
HAGOS	.916
HOOS	.881
SC-iHOT-33	.732
QPFPTHA	.832

Copenhagen Hip and Groin Outcome Score (HAGOS); International Hip Outcome Tool (SC-iHOT-33); Osteoarthritis of Knee and Hip Quality of Life (OAKHQOL); Oxford Hip Score (OHS); Hip disability and Osteoarthritis Outcome Score (HOOS); Questionnaire on the Perceptions and Functions of Patients about Total Hip Arthroplasty (QPFPTHA);

## Discussion

Global EMPRO scores from the assessment exercise indicated two PROMs had total scores above threshold, with the highest being OAKHQOL, followed by HAGOS. Four instruments could not be scored for cultural and language adaptation and differential item functioning and harmonization was often under documented.

Reliability, validity and responsiveness properties all had scores greater than threshold except for QPFPTHA. We found that these domains were more thoroughly evaluated than the others. Although interpretability is not a measurement property as it does not refer to the inherent quality of an instrument, it is a measure of practicality for clinical and research use. This property often receives less attention in studies of instrument quality [19]. All hip PROMs had limited data on this attribute and scores could not be assigned.

Except for OHS which had two studies on measurement properties, all other assessed hip PROMS were supported by a single paper. Opportunities for gaining improved scores may have been restricted by the limited data set for analysis. More holistic assessments can be made where several articles describe attribute data which can be pooled [17].

Arthroplasty registries use PROMs because recording a high rate of technical success after arthroplasty does not capture the variability in pain and function experienced by patients [12]. In 2016, up to 18 national arthroplasty registries used PROMs which are published in their reports [29]. Rolfson et al [30], identified that hip-specific PROMs used in arthroplasty registries include OHS, HOOS, HOOS short form (joint replacement), Harris Hip Score (strictly not a PROM since it has a clinician-assessed component), Western Ontario and McMaster Universities Arthritis Index (WOMAC) and University of California at Los Angeles (UCLA) Activity Score. According to the International society of Arthroplasty Registries (ISAR) Working Group there is no single best generic or hip-specific PROM [12] however it is self-evident that PROMs have to be developed in appropriate patient population and have good measurement properties. Measurements of quality have varied between researchers, for example with the OHS [15, 31]. Some of these differences seem to be due to the availability of new evidence in later publications, reflecting a dynamic process of accumulating evidence of PROM utility.

In this study, reviewers were able to recommend all evaluated PROMs, but *‘with provisos and alterations’*. Among these, only OHS and HOOS are commonly used hip-specific PROMs in registries worldwide. However, these failed to achieve threshold for acceptability using EMPRO scoring criteria. We identified HAGOS and OAKHQOL as having acceptable global scores. However, HAGOS is designed specifically for younger individuals with hip disease, and OAKHQOL is not commonly used in arthroplasty registries [30]. Chinese patient groups used to test the culturally-adapted PROMs mainly had osteoarthritis, although rheumatoid arthritis, avascular necrosis and fracture patients were included. All studies which tested responsiveness used arthroplasty as the intervention. All cohorts were younger than might be expected in arthroplasty patient groups elsewhere; mean age of each cohort in this series ranged from 43 to 66 years (Table 1).

Limitations of this study include that it relies on a single instrument (EMPRO) to assess measurement qualities semi-quantitatively, so the weighting of attribute and items within attributes has certain subjective features; however, this study is the first assessment of cross culturally adapted Chinese-language hip PROMs within a dynamic evaluative process. We used two experienced reviewers; more would have added further reliability to the assessment, however they achieved good-excellent ICC scores for inter-rater agreement.

## Recommendations and conclusions

Among the commonly used hip-specific PROMs found in arthroplasty registries, none of the Chinese adapted versions evaluated by EMPRO is currently rated acceptable for clinical use. Compared to other non-English language environments which have developed PROM initiatives such as BiblioPro, a Spanish language-based PROM online library [32], there are none presently in China. We recommend that further studies on measurement qualities of these PROMs should remain a priority and include older patient groups. Deficient domains include cultural and language adaptation, interpretability and burden. WOMAC and UCLA Activity Score are commonly found in registries, and have been culturally adapted for Chinese patients with knee osteoarthritis but not for the hip [33, 34]. Since Chinese patients face barriers to follow-up after surgery due to large travel distances and lack of resources, the properties of electronic or telephonic methods of administration should also receive attention. Similar to the conclusion of researchers in other non-English language settings [14], we also cannot recommend any single hip-specific Chinese-mainland adapted PROM at present.

## Supporting information

S1 Checklist PRISMA 2009 checklist. (DOC)

S1 File Pubmed/Medline filter: Psychometric properties of specific PROMs questionnaires in the Chinese population. (PDF)
